# Recurrent Infection-Triggered Immune Thrombocytopenia Flares in Medullary Sponge Kidney: A Case Report

**DOI:** 10.7759/cureus.114505

**Published:** 2026-08-13

**Authors:** Mahmoud Alothman Agha, Shuaib Alsibai, Laith Suwan, Ahmed M Saleh, Ruchik Kevadiya, Adam Fletcher

**Affiliations:** 1 Medicine, Mediclinic, Abu Dhabi, ARE; 2 Internal Medicine, Independent Research, Chicago, USA; 3 Medicine, Al Garhoud Private Hospital, Dubai, ARE; 4 Internal Medicine, Wayne State University School of Medicine, Detroit, USA; 5 Internal Medicine, Henry Ford Rochester Hospital/Wayne State University, Rochester Hills, USA

**Keywords:** hematuria, immune thrombocytopenia, intravenous immunoglobulin, medullary sponge kidney, nephrolithiasis, pyelonephritis, recurrent urinary infections, ureteral stenting

## Abstract

Medullary sponge kidney (MSK) is a congenital collecting duct abnormality associated with nephrocalcinosis, recurrent nephrolithiasis, hematuria, urinary tract infections, and obstructive urological complications. Immune thrombocytopenia (ITP) is an acquired autoimmune disorder characterized by isolated thrombocytopenia and bleeding risk. We report a case illustrating how these two chronic conditions can interact indirectly to create a recurrent management dilemma.

A 45-year-old woman with bilateral MSK and chronic ITP controlled with weekly romiplostim had a baseline platelet count of approximately 120-130 × 10³/µL. Over several months, she experienced recurrent urological events, including obstructive nephropathy, extended-spectrum beta-lactamase (ESBL) urosepsis, pyelonephritis, ureteritis, and repeated ureteral stenting. During a March 2026 admission for right pyelonephritis with bilateral stents, her platelet count fell abruptly from approximately 120 × 10³/µL to 7 × 10³/µL, accompanied by gross hematuria, and improved after intravenous immunoglobulin and dexamethasone. She presented again in April 2026 with left flank pain, nausea, gross hematuria, leukocytosis, positive urinalysis, bilateral ureteral wall thickening, periureteral stranding, mild left hydronephrosis, and bilateral medullary calcifications consistent with MSK. Her platelet count had again declined to 27 × 10³/µL. Workup did not support thrombotic microangiopathy, disseminated intravascular coagulation, pseudothrombocytopenia, drug-induced thrombocytopenia, hemolysis, viral infection, or renal failure as alternative causes. She was treated with meropenem, fluconazole, hydration, analgesia, and intravenous immunoglobulin, while corticosteroids were deferred because of active infection and romiplostim was held. Platelets rose to 116-125 × 10³/µL within 48 hours, with resolution of hematuria and clinical improvement.

This case emphasizes that recurrent MSK-related urinary infections may precipitate clinically significant ITP flares. Early platelet monitoring and coordinated hematology, infectious disease, and urology input are essential when infection control, bleeding risk, and procedural decisions overlap.

## Introduction

Medullary sponge kidney (MSK) is an uncommon congenital abnormality of the medullary collecting ducts associated with nephrocalcinosis, recurrent nephrolithiasis, hematuria, and urinary tract infection [1,2]. Estimates of its prevalence range from approximately 1 in 5,000 to 1 in 20,000 people, with a substantially higher frequency among recurrent calcium stone formers [1,2]. 

Immune thrombocytopenia (ITP) is an acquired immune-mediated disorder characterized by isolated thrombocytopenia and bleeding risk, with an estimated adult incidence of approximately 1.6-3.9 per 100,000 person-years [3]. The frequency of coexisting MSK and ITP has not been established, and to our knowledge, this combination has not previously been described as a recurrent clinical-management problem in which MSK-associated urologic infection is temporally associated with repeated ITP flares. This case illustrates how recurrent stone disease, instrumentation, and infection in MSK may destabilize otherwise controlled chronic ITP and complicate bleeding and procedural management.

## Case presentation

A 45-year-old woman with known bilateral MSK, diagnosed in her 20s, and chronic ITP of more than one year’s duration presented following a series of recurrent stone- and infection-related urologic admissions. Her ITP had been managed with weekly romiplostim, maintaining a usual platelet count of approximately 120-130 × 10³/µL. Her medical history was also notable for hypertension treated with amlodipine, recurrent nephrolithiasis requiring multiple lithotripsies, and previous ureteral stent placements. A prior 24-hour urine metabolic stone evaluation demonstrated hypercalciuria and hypocitraturia. She had not undergone splenectomy and had no other documented autoimmune disease.

Approximately four months before the index admission, she required left ureteral stenting for obstructive nephropathy without a documented ITP flare. Approximately three months before admission, she developed extended-spectrum beta-lactamase (ESBL)-producing *Escherichia coli* urosepsis, which was treated with broad-spectrum antibiotics, while her platelet count remained near baseline. Approximately one month before admission, she was hospitalized with right-sided pyelonephritis in the presence of bilateral ureteral stents. During that episode, her platelet count declined from approximately 120 × 10³/µL to 7 × 10³/µL and was accompanied by gross hematuria. She received intravenous immunoglobulin and intravenous dexamethasone, with platelet recovery to 28 × 10³/µL by discharge. This recurrent pattern suggested that urologic infections could precipitate abrupt and clinically significant exacerbations of her chronic ITP.

On hospital day 1 of the index admission, she presented with a two-day history of left-sided flank pain, nausea, and gross hematuria after passing gravel-like stone fragments the previous night. A short course of oral cefuroxime, prescribed one week earlier for a presumed urinary tract infection, had not improved her symptoms. She denied fever, chills, recent vaccination, or exposure to new medications. On examination, she was afebrile, with a blood pressure of 148/92 mmHg and a heart rate of 96 beats/min. She appeared uncomfortable and had costovertebral angle tenderness that was more pronounced on the left. There was no rash, petechiae, mucosal bleeding, hepatosplenomegaly, or lymphadenopathy.

Laboratory findings (Table 1) demonstrated neutrophilic leukocytosis, chronic microcytic anemia, and severe thrombocytopenia representing an abrupt decline from her usual romiplostim-supported baseline. Urinalysis showed marked pyuria, hematuria, and findings suggestive of urinary tract infection. Renal function was preserved, while coagulation studies, hemolysis markers, and liver enzymes were within normal limits. Peripheral blood smear confirmed marked thrombocytopenia with large platelets and showed no schistocytes or blasts, while repeat testing using a citrated sample excluded pseudothrombocytopenia.

**Table 1:** Laboratory findings at admission

Laboratory test	Patient result	Reference range	Unit	Interpretation
White blood cell count	18	4.0-11.0	×10³/µL	Elevated
Neutrophils	86	40-70	%	Elevated
Hemoglobin	10.6	12.0-15.5	g/dL	Reduced
Platelet count	27	150-450	×10³/µL	Markedly reduced
Serum creatinine	0.6	0.5-1.1	mg/dL	Normal
Estimated glomerular filtration rate	>100	≥90	mL/min/1.73 m²	Preserved renal function
Prothrombin time	12	11-13.5	Seconds	Normal
International normalized ratio	0.9	0.8-1.1	Ratio	Normal
Activated partial thromboplastin time	28	25-35	Seconds	Normal
Fibrinogen	230	200-400	mg/dL	Normal
Lactate dehydrogenase	160	140-280	U/L	No biochemical evidence of hemolysis
Haptoglobin	75	30-200	mg/dL	No biochemical evidence of hemolysis
Liver enzymes	Within normal limits	Laboratory-specific	U/L	Normal
Urine blood	Large	Negative	Qualitative	Positive
Urine white blood cells	>100	0-5	Cells/high-power field	Marked pyuria
Urine red blood cells	30-50	0-2	Cells/high-power field	Hematuria
Urine leukocyte esterase	Positive	Negative	Qualitative	Suggestive of urinary tract inflammation/infection
Urine nitrite	Positive	Negative	Qualitative	Suggestive of nitrate-reducing bacteria
Peripheral blood smear (platelets)	Marked thrombocytopenia with large platelets	Adequate number with normal morphology	Qualitative	Consistent with peripheral platelet destruction
Peripheral blood smear (schistocytes)	Absent	Absent	Qualitative	No morphological evidence of microangiopathic hemolysis
Peripheral blood smear (blasts)	Absent	Absent	Qualitative	No circulating blasts identified
Platelet count in citrated sample	Confirmed true thrombocytopenia; exact value not reported	150-450	×10³/µL	Pseudothrombocytopenia excluded

**Figure 1:**
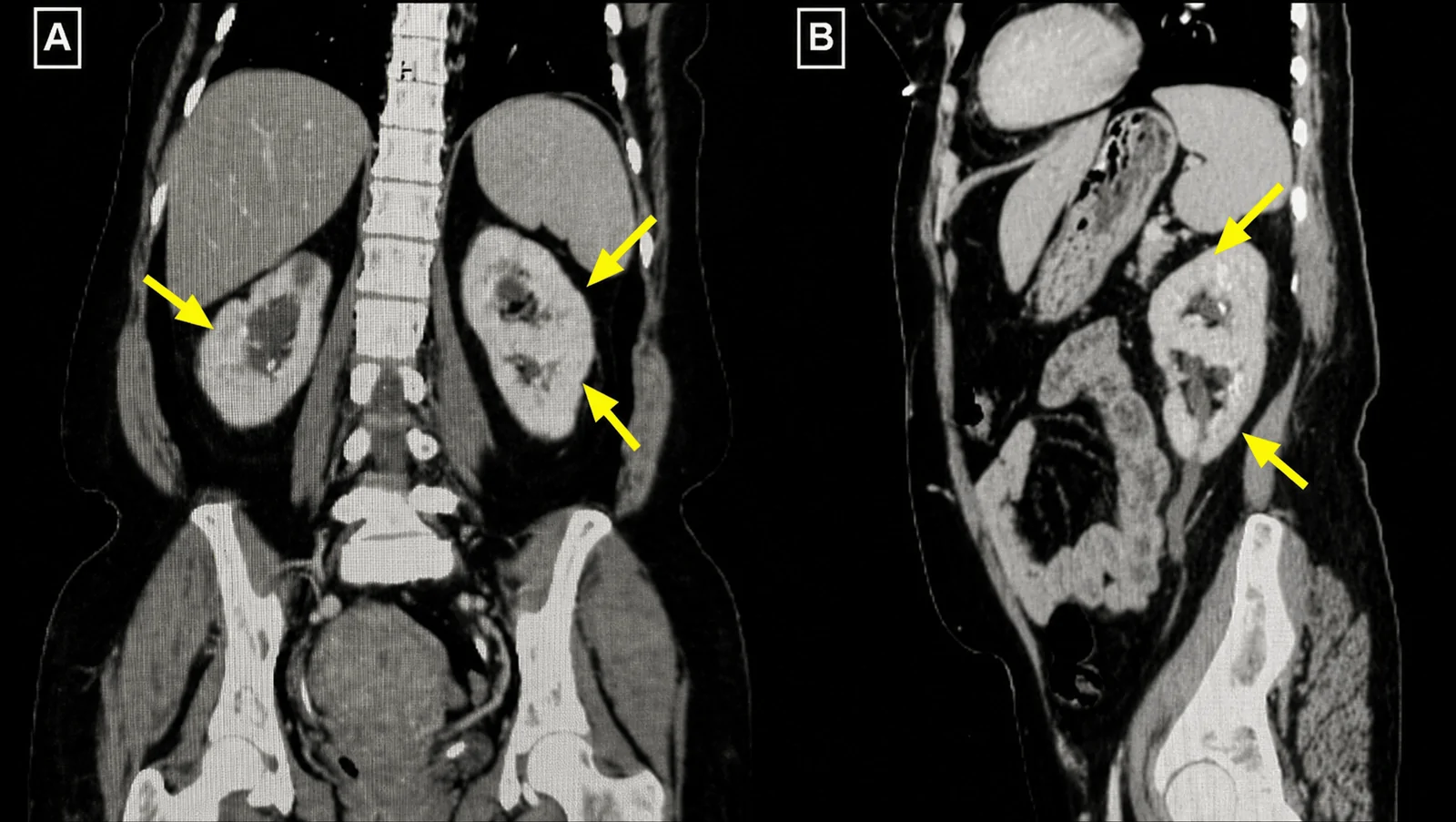
Contrast-enhanced CT of the abdomen and pelvis (A) Coronal image demonstrating bilateral medullary nephrocalcinosis with numerous calcifications concentrated in the renal pyramids, small non-obstructing renal calculi, mild left hydronephrosis, and partially visualized bilateral ureteral stents. (B) Sagittal image through the left kidney demonstrating mild pelvicalyceal dilatation and extensive medullary calcifications consistent with medullary sponge kidney. Bilateral ureteral wall thickening and periureteral fat stranding on the complete examination were consistent with ureteritis/pyelonephritis.

Contrast-enhanced computed tomography of the abdomen and pelvis confirmed bilateral MSK with chronic stone disease and indwelling ureteral stents, accompanied by mild left hydronephrosis and inflammatory changes compatible with ureteritis/pyelonephritis. The detailed radiologic findings are shown in Figure 1.

She was evaluated jointly by hematology, infectious disease, and urology and was started on intravenous meropenem, intravenous fluconazole, hydration, analgesia, and intravenous immunoglobulin at 1 g/kg/day for two days. Romiplostim was temporarily withheld, and corticosteroids were deferred because of the active infection. Urine culture grew *Candida *species, interpreted as likely colonization of the chronic ureteral stents. One blood culture set yielded gram-positive cocci in clusters; however, this was considered contamination because repeat cultures were negative. Human immunodeficiency virus and hepatitis B and C serologies were negative. The overall clinical picture was most consistent with complicated bilateral ureteritis/pyelonephritis associated with chronic ureteral stenting and a concomitant infection-triggered exacerbation of chronic ITP. Alternative causes of thrombocytopenia were not supported, as there was no evidence of thrombotic microangiopathy, disseminated intravascular coagulation, new drug exposure, renal failure, or pseudothrombocytopenia.

By hospital day 2, following intravenous immunoglobulin and antimicrobial treatment, her platelet count had increased rapidly to approximately 116 × 10³/µL, with improvement in hematuria and infectious symptoms. By hospital day 3, the platelet count had stabilized at approximately 125 × 10³/µL, gross hematuria had resolved, and her overall clinical condition had continued to improve. She was subsequently discharged on oral cefuroxime with planned hematology and urology follow-up, including evaluation of the indwelling ureteral stents. The relationship between the patient’s recurrent urologic and infectious episodes and the corresponding platelet-count fluctuations is summarized in Table 2.

**Table 2:** Timeline of urologic infections and associated platelet count trends relative to the index admission ESBL: extended-spectrum beta-lactamase, ITP: immune thrombocytopenia.

Time relative to index admission	Urologic/infectious event	Microbiological finding	Pre-event platelet count	Lowest documented platelet count	Post-treatment/recovery
Approximately 4 months before admission	Left obstructive nephropathy requiring ureteral stenting	Not reported	~115 × 10³/µL	No documented ITP flare	Not reported
Approximately 3 months before admission	Urosepsis	ESBL-producing Escherichia coli in urine	~130 × 10³/µL	Stable at approximately 120 × 10³/µL	~120 × 10³/µL
Approximately 1 month before admission	Right pyelonephritis in the presence of bilateral ureteral stents	Candida species in urine	~120 × 10³/µL	7 × 10³/µL	28 × 10³/µL at discharge
Hospital day 1	Bilateral ureteritis/pyelonephritis with indwelling ureteral stents	Urine culture grew Candida species; one blood culture yielded gram-positive cocci considered a contaminant	~130 × 10³/µL	27 × 10³/µL	Intravenous immunoglobulin and antimicrobial therapy initiated
Hospital day 2	Continued treatment of infection-triggered ITP exacerbation	Repeat blood cultures were negative	-	116 × 10³/µL	Hematuria and infectious symptoms improved
Hospital day 3/discharge	Continued clinical recovery	-	-	125 × 10³/µL	Platelet count stabilized, hematuria resolved, and the patient was discharged

## Discussion

Literature relevance and clinical novelty

To our knowledge, the coexistence of MSK and chronic ITP has not been previously described as a recurrent clinical management dilemma. MSK is a congenital abnormality characterized by cystic dilatation of the medullary and papillary collecting ducts and is typically diagnosed radiologically. Although often benign, symptomatic MSK is strongly associated with nephrocalcinosis, recurrent nephrolithiasis, hematuria, urinary tract infections, and, in some cases, distal renal tubular acidosis [4,5]. In contrast, ITP is an acquired immune-mediated thrombocytopenic disorder characterized by isolated thrombocytopenia due to accelerated platelet destruction and/or impaired platelet production, and its diagnosis relies on excluding alternative causes of thrombocytopenia [6,7].

The relevance of this case is therefore not a proposed direct pathophysiologic association between MSK and ITP, but rather the clinically important interaction between two chronic conditions. In this patient, MSK created a recurrent urologic substrate of nephrolithiasis, obstruction, ureteral stenting, and complicated urinary infection. These infectious and inflammatory episodes were temporally associated with repeated destabilization of chronic ITP, producing abrupt platelet declines and hematuria. This interaction created a recurring diagnostic and therapeutic challenge: hematuria could be attributed to stone disease, infection, or thrombocytopenia; urologic source control or stent manipulation had to be balanced against bleeding risk; and ITP-directed therapy had to be selected carefully in the setting of active infection.

MSK as a recurrent urological and infectious substrate

Although many patients remain asymptomatic, symptomatic MSK is commonly associated with nephrocalcinosis, recurrent nephrolithiasis, hematuria, urinary tract infections, and metabolic abnormalities such as hypocitraturia, hypercalciuria, and distal renal tubular acidosis. These abnormalities promote urinary stasis and stone formation, predisposing affected patients to recurrent renal colic, obstruction, infection, and, in selected cases, repeated endourological interventions for stone-related complications [8].

In our patient, MSK appeared to be the underlying condition driving her recurrent urological burden. She had a long history of bilateral nephrocalcinosis and recurrent nephrolithiasis requiring multiple lithotripsies and ureteral stent placements, followed by several recent admissions for obstructive nephropathy, pyelonephritis, and ureteritis. The combination of recurrent stone disease, obstruction, and chronic indwelling ureteral stents likely created a persistent substrate for urinary tract inflammation and infection. Therefore, although MSK is not itself a hematologic disorder, it indirectly contributed to clinically significant hematologic instability by generating recurrent infectious and inflammatory episodes that precipitated exacerbations of her chronic ITP.

Recurrent infection-triggered ITP flares

A central observation in this case was the repeated temporal association between complicated urinary infection and acute exacerbation of chronic ITP. The recurrence of this pattern across sequential admissions supports the interpretation that urinary infection and stent-related inflammation acted as clinically relevant triggers for ITP destabilization.

The biological plausibility of this relationship is supported by current understanding of ITP pathogenesis. ITP involves a complex interaction of platelet autoantibodies, Fc receptor-mediated platelet clearance by macrophages, impaired platelet production, complement activation, and T-cell dysregulation [9-11]. Acute infection may amplify these immune pathways through systemic inflammation, cytokine release, innate immune activation, and immune cross-reactivity, thereby worsening platelet destruction in patients with pre-existing auto-ITP [9-11]. In this patient, the urinary infections likely did not cause de novo ITP; rather, they appear to have precipitated flares of an established chronic ITP that had previously been controlled with romiplostim.

The urinary tract context is also clinically relevant. Indwelling urinary devices, including ureteral stents, can become colonized and support biofilm formation, which may contribute to persistent bacteriuria, candiduria, recurrent urinary inflammation, and difficult-to-eradicate infection [12,13]. Therefore, the novelty of this case lies not in proposing a direct shared pathogenesis between MSK and ITP, but in demonstrating how a structural renal disorder with recurrent stone disease, obstruction, stenting, and infection can indirectly destabilize an autoimmune hematologic condition. This interaction transformed recurrent urological episodes into repeated hematologic emergencies and created a recurring need to balance infection control, bleeding risk, and ITP-directed therapy.

Management dilemma and multidisciplinary approach

This case highlights the complexity of managing complicated urinary infection in a patient with MSK and coexisting chronic ITP. The principal management challenge was the simultaneous presence of pyelonephritis/ureteritis, gross hematuria, and severe thrombocytopenia. In patients with recurrent infection and chronic ureteral stenting, urological intervention or stent exchange may be required for source control; however, severe thrombocytopenia substantially increases the risk of peri-procedural bleeding. In this context, rapid platelet correction was necessary before considering any invasive urological intervention.

Intravenous immunoglobulin was selected because a rapid increase in platelet count was desired in the setting of active bleeding and severe thrombocytopenia. IVIG is recommended in urgent ITP scenarios when a prompt but temporary platelet rise is required, including severe bleeding or preparation for invasive procedures [14]. In our patient, platelet recovery occurred within 24-48 hours after IVIG administration, accompanied by resolution of hematuria. Corticosteroids, although commonly used in ITP, were minimized during the April admission because the patient had active pyelonephritis, and high-dose glucocorticoids may increase infection risk through dose-dependent immunosuppressive effects [15].

Optimal management required close multidisciplinary collaboration. Hematology guided the acute ITP flare management with IVIG and temporary withholding of romiplostim; infectious disease directed antimicrobial therapy in the setting of complicated pyelonephritis and prior resistant organisms; and urology evaluated the need for further stent-related intervention while balancing the risk of bleeding. This coordinated approach allowed simultaneous treatment of infection and thrombocytopenia while avoiding unnecessary procedural risk during the period of greatest hematologic instability.

## Conclusions

This case highlights a clinically important interaction between MSK and chronic ITP, in which recurrent stone and infection-related urologic episodes appeared to precipitate abrupt ITP flares with bleeding risk. Although a direct pathophysiologic association cannot be inferred, MSK created a persistent substrate for recurrent urinary infection, obstruction, and stent-related inflammation that repeatedly complicated hematologic stability. The case underscores the need for early recognition of infection-triggered thrombocytopenic deterioration, prompt treatment of the underlying urologic infection, careful selection of ITP-directed therapy during active infection, and close collaboration between hematology, urology, and infectious disease teams. Clinicians managing patients with chronic ITP and recurrent urologic disease should monitor platelet trends closely during infectious episodes and incorporate infection control and urologic source management into relapse prevention. The patient continues under ongoing multidisciplinary follow-up.
